# Exploring posterosuperior bundle area pacing: A novel atrial pacing modality with efficient anatomical access, stable pacing lead fixation, and reserved interatrial synchrony

**DOI:** 10.1016/j.hroo.2024.11.027

**Published:** 2024-12-05

**Authors:** Zhaohui Qiu, Xianhao Wu, Wei Hu, Jing Ni, Zhongping Yang, Hongyang Lu

**Affiliations:** 1 Department of Cardiology, Shanghai Tong Ren Hospital, Shanghai Jiao Tong University School of Medicine, Shanghai, China; 2 Cardiac Rhythm Management, Medtronic, Mounds View, Minnesota; 3 Cardiac Rhythm Management, Medtronic Technology Center, Medtronic, Shanghai, China

**Keywords:** Posterosuperior bundle area pacing, Bachmann's bundle pacing, Atrial pacing, Interatrial synchrony, Superior vena cava, Case report

**Key Findings**

▪ This case report describes 2 cases of dual-chamber pacing in which Bachmann's bundle was initially targeted, but both ultimately resulted in successful pacing in the posterosuperior bundle (PSB) area within the superior vena cava, as verified by follow-up computed tomography imaging.
▪ Sensing amplitude in the PSB area showed a good electrical engagement, while a split electrogram indicated a possible potential at the PSB. However, the anatomical location is the key point of the pacing lead tip position, utilizing efficient anatomical access in the PSB area.
▪ Follow-up results at 11 and 9 months after PSB implantation showed stable electrical parameters with no perforation observed, and pulsed Doppler measurements in 1 case showed reserved interatrial synchrony.
▪ Further clinical research is necessary to investigate the electrical and anatomical characteristics of the PSB, its electrical and anatomy-involved capture criteria, and long-term reliability and clinical outcomes.

Atrial pacing has developed into a commonly adopted modality in the right atrial appendage (RAA), which has remained unchanged since its introduction decades ago. Bachmann's bundle (BB) pacing is an alternative to RAA pacing, which is currently being studied.^1^ In this article, we describe 2 consecutive cases in which we initially targeted BB and utilized atrial sensing amplitude as the electrical marker; however, both ultimately resulted in successful pacing in the posterosuperior bundle (PSB) area, demonstrating a novel atrial pacing modality within the superior vena cava (SVC), superior and posterior to the level of BB verified by follow-up computed tomography (CT) imaging, in which the pacing lead could efficiently access during implantation, and provided stable fixation and reserved interatrial synchrony at 11- and 9-month follow-ups.

## Case 1

A 55-year-old female with high-grade atrioventricular block presented in August 2023. She experienced severe dizziness for a month with symptoms of syncope. Echocardiography reported normal left ventricular ejection fraction (67%), left atrial diameter (37 mm), left ventricular end-diastolic diameter (44 mm), and left ventricular end-systolic diameter (28 mm). Mild to moderate tricuspid regurgitation was observed. Electrocardiography (ECG) and a prior implanted insertable cardiac monitor (Reveal LINQ; Medtronic) showed an intermittent high-grade atrioventricular block, with a P-wave width of 118 ms and QRS duration of 86 ms. Based on the findings, and in an effort to maintain atrial and ventricular synchrony, implantation of a dual-chamber pacemaker with BB and left bundle branch (LBB) pacing was planned.

**Figure 1:**
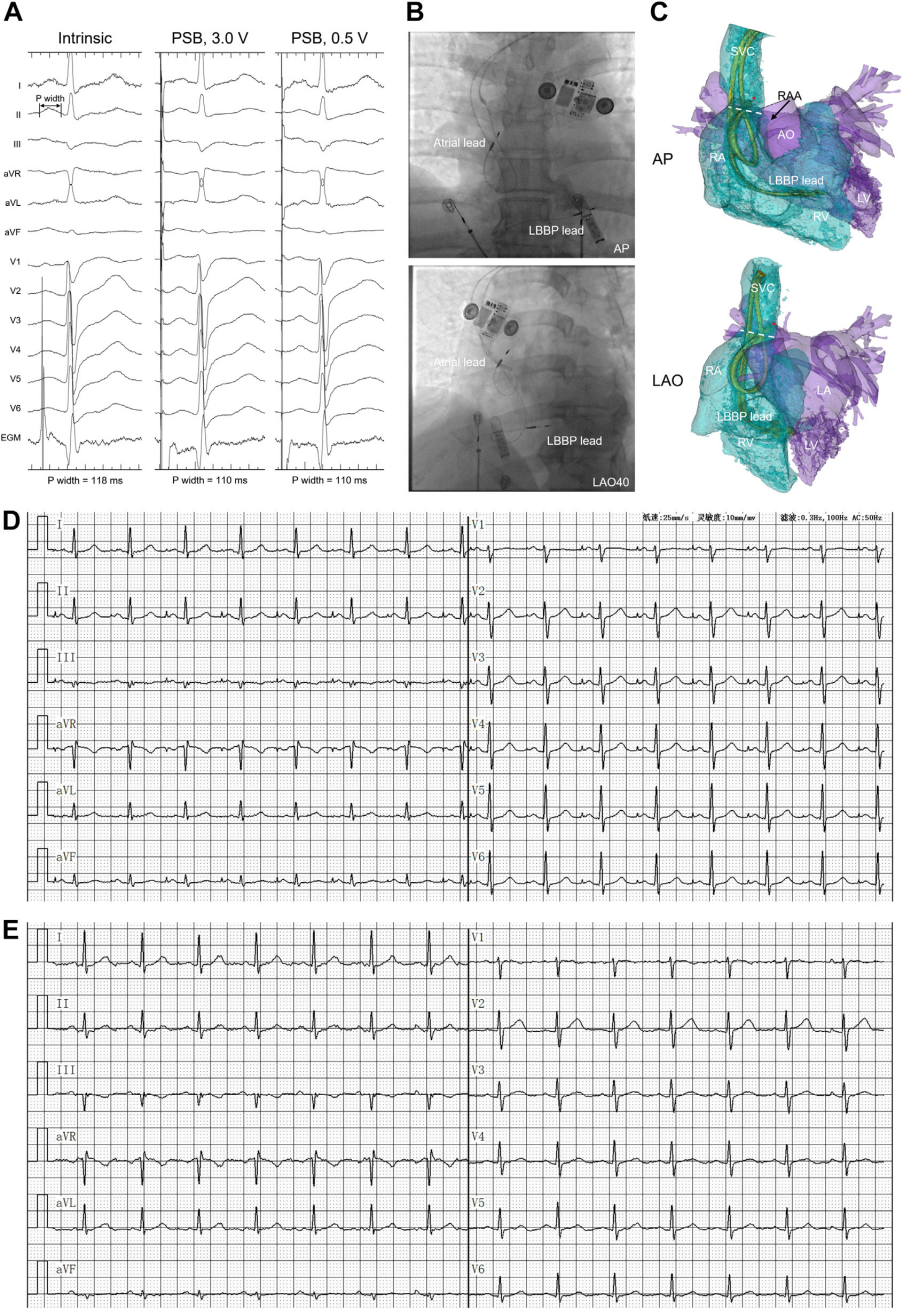
(A) Twelve-lead electrocardiogram (ECG) and intracardiac electrogram (EGM) at the posterosuperior bundle (PSB) location showing a narrow P-wave with similar P-wave morphology between intrinsic and paced waveforms. (B) Fluoroscopic images in 2 views of the pacing lead located at the PSB location. (C) Computed tomography reconstruction result showing the pacing lead fixed in the posteromedial wall of the superior vena cava (SVC) superior to the junction of SVC and right atrium (RA). The asterisk indicates the PSB location; the dashed line indicates the SVC/RA junction. (D) Atrial-paced ECG recorded at 11-month follow-up. (E) ECG with intrinsic P waves at follow-up. AO = aorta; AP = anteroposterior; LA = left atrium; LAO = left anterior oblique; LBBP = left bundle branch pacing; LV = left ventricle; RA = right atrium; RAA = right atrial appendage; RV = right ventricle.

After obtaining axillary venous access, a 3830 pacing lead (SelectSecure; Medtronic) was screwed into the interventricular septum and reached the LBB through a delivery catheter (Model C315His; Medtronic). BB pacing was then attempted with a second 3830 pacing lead and another delivery catheter (Model C315S4; Medtronic). The catheter was advanced under the right anterior oblique 30° in the right atrium (RA) as far cranially as possible to approach the roof of the RA and rotated counterclockwise toward the interatrial septum. To locate BB, the catheter was pointed leftward under the left anterior oblique 40° view, and the catheter tip was seen superior to the base of the auricle under right anterior oblique 30°, pointing superoanteriorly toward the aorta. In our routine practice during LBB pacing lead implantation, R-wave sensing amplitude has been utilized as an electrical marker in the endocardium to locate an ideal target area in the ventricle; therefore, we located the pacing lead with the purpose of searching for a BB location with desirable atrial sensing amplitudes. Specifically, we assessed the locations while adjusting the catheter to move around the pacing lead tip superiorly, as BB passes the interatrial groove in the superior. At the final location we assessed, the atrial sensing amplitude was 1.5 mV. With unipolar atrial pacing, positive P waves were presented in leads I and II; the capture threshold was 0.5 V with a pulse width of 0.5 ms, and the paced P-wave width was 110 ms. Intervals from the pacing spike to P-wave onset and QRS onset were seen (35 ms and 170 ms) (Figure 1A). Three or four turns were then applied to the pacing lead for fixation (Figure 1B).

To verify the fixation of the pacing lead, a CT scan was conducted during the 1-month postimplantation follow-up. The result suggested that the pacing lead tip was unintentionally placed in the area where the PSB extended around the SVC.^2^, ^3^, ^4^ Specifically, the tip was located in the posteromedial wall of the SVC and fixed at the SVC muscle sleeve between the caval sphincter-like muscle and the SVC/RA junction (Figure 1C).

At the 11-month follow-up, echocardiography reported normal left ventricular ejection fraction (66%), left atrial diameter (36 mm), left ventricular end-diastolic diameter (45 mm), left ventricular end-systolic diameter (29 mm), and mild tricuspid regurgitation. ECG measurements showed a paced P-wave width of 100 ms and QRS duration of 92 ms with PSB and inhibited LBB pacing (Figures 1D and 1E). The atrial sensing amplitude was 1.8 mV and the capture threshold remained 0.75 V with a pulse width of 0.4 ms. Pulsed Doppler measurements showed simultaneous start of left atrial and RA contraction with a minimal time difference (<5 ms) observed between the initiation of A waves in the flow velocity plots at the tricuspid and mitral valves. The patient had no hospitalizations since the implantation, and no pacing lead dislodgement or perforation was observed.

## Case 2

**Figure 2:**
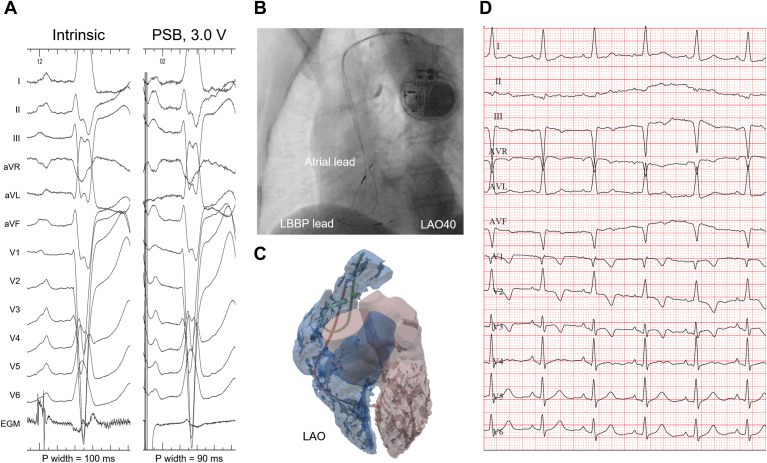
(A) Twelve-lead electrocardiogram (ECG) and intracardiac electrogram of intrinsic and posterosuperior bundle (PSB) paced waveforms. (B) Fluoroscopic images in left anterior oblique 40° (LAO40) view of the pacing lead located at the PSB area. (C) Computed tomography reconstruction result showing the pacing lead fixed in the posteromedial wall of the superior vena cava superior to the junction of the superior vena cava and right atrium. (D) Paced ECG recorded at 9-month follow-up. LBBP = left bundle branch pacing.

Another case of PSB area pacing involves a 77-year-old female indicated for dual-chamber pacemaker implantation who presented in October 2023. ECG showed complete atrioventricular block, complete right bundle branch block, and frequent premature atrial contraction, with a ventricular rate of 50 beats/min, P-wave width of 100 ms, P-wave axis of 18°, and QRS duration of 130 ms (Figure 2A). LBB pacing was first conducted with the same procedure. At the final atrial pacing location (Figure 2B), the atrial sensing amplitude was 2.5 mV, a split electrogram (EGM) was observed at the upstroke of the P waves; the capture threshold was 1.3 V with a pulse width of 0.5 ms, and the paced P-wave width was 94 ms. Ten days after the implantation, CT results confirmed PSB area pacing (Figure 2C). At the 9-month follow-up, ECG measurements showed a paced P-wave width of 90 ms, P-wave axis of 58°, and QRS duration of 104 ms with LBB pacing (Figure 2D). The atrial sensing amplitude was 1.2 mV and the capture threshold was 0.75 V with a pulse width of 0.4 ms. Similarly, the patient had no hospitalizations since the implantation, and no pacing lead dislodgement or perforation was observed.

## Discussion

Successful capture of the PSB is suggested by (1) fixation of a pacing lead in the posteromedial wall of the SVC superior to the SVC/RA junction where the PSB extends into the SVC; (2) a narrower paced P-wave width compared with conventional RAA pacing, with similar P-wave morphology but similar or longer paced PR interval to those of the intrinsic rhythm; and (3) an adequate atrial sensing amplitude. The PSB is a distinct interatrial connection that runs posterior to the level of BB and joins the BB from the posterior border over the interatrial groove, extending to the RA and enveloping the SVC wall.^2^^,^^3^ Therefore, pacing at the PSB area may produce a similar electrical activation pattern to BB pacing, which is considered an atrial physiological pacing modality. An earlier study revealed that the PSB was present in all examined hearts.^4^ Furthermore, anatomic studies have demonstrated the lateral existence of the SVC muscle sleeve that extends 2 to 5 cm from the RA,^5^ with a thickness of 1.2 ± 1.0 mm within the venous wall,^6^ potentially allowing for effective pacing stimuli. This case report suggests that differentiating PSB pacing from BB pacing could be based on the anatomical marker under angiographic observations, as we believe that the anatomy of the SVC and the PSB provides a distinct foundation of direct access and a repeatable process for the pacing lead, compared with other approaches, in terms of identifying, mapping, and locating the appropriate pacing location.

These cases report PSB area pacing in clinical practice for the first time. Follow-up results at 11 and 9 months after implantation showed stable electrical parameters, such as pacing capture threshold and atrial sensing amplitude. Clinical outcomes also showed reserved interatrial synchrony partly suggested by the synchronized activity of tricuspid and mitral valves. During the entire follow-up period, no perforation was observed in either case; however, the anatomy of the PSB area, particularly the thickness of the targeted pacing location, requires further evaluation.

Electrical mapping was useful as sensing amplitude indicated a good electrical engagement in the PSB area on the SVC subendocardium (ie, the inner layer), letting the pacing lead access the desirable pacing location directly. In this case, the CT reconstruction result showed that no obvious boundaries existed between the RA posterior wall and the SVC, in line with the previous observation,^7^ which helped the search for the location with a desirable atrial sensing amplitude and ultimately resulted in the unintentional placement of the pacing lead tip in the PSB area. Therefore, fluoroscopy with contrast media is recommended for verifying the anatomy around the PSB area.

A split EGM was observed at the upstroke of the P waves in 1 of the 2 cases (Figure 2A), indicating a possible potential at the PSB that represented the local electrical activities of the neighboring atrial myocardium. Unlike the His bundle or LBB, the internodal or interatrial bundles, including BB, are not insulated by a surrounding fibrous sheath but instead are insulated by multiple anisotropically structured atrial myocardial conduction connections (ie, muscle bundles).^8^ As a result, the potential of BB or the PSB may not be constantly observed or indicative of bundle capture during the procedure. This implies less significance of the potentials of the atrial bundles than the combined criteria that emphasize anatomic markers for PSB pacing, with the latter offering an advantage that distinguishes PSB pacing from other approaches under fluoroscopy, although the application of the split EGM as one of the criteria needs to be verified in further investigations.

BB pacing is an alternative to RAA pacing, which is currently being developed. Studies have shown that it can result in a narrower or comparable P-wave width compared with sinus rhythm and may reduce the risk of atrial fibrillation,^9^^,^^10^ although the investigation results were inconsistent.^10^ Despite these promising findings, BB pacing has yet to be widely adopted in clinical practice due to 3 barriers: (1) the small size of the BB area in the RA epicardium makes it challenging to map and locate pacing leads; (2) the anatomical marker and electrical capture criteria to prove a BB capture are unclear; and (3) the anatomical characteristics of the atrial wall at the BB area, including the smooth and flat endocardium in most patients, require extensive procedure and fluoroscopy to fix pacing leads. Anatomical location is the key point of the lead tip position, while PSB is anatomically near the right superior pulmonary vein, and the BB area is close to the aorta.^4^ Recently, BB pacing was assessed in the neighborhood of the high RA septum below the location of the PSB, with more concise electrical criteria.^11^ However, this case report raises the question of how electrical criteria apply for atrial bundle capture given the comprehensive myocardial connections in the atrial wall, resulting in uncharacteristic electrical activities or similar responses (eg, P-wave shortening and presence of local electrical potential) to pacing at different locations across relatively wide areas within the RA. In the quest for atrial physiological pacing, with further clinical investigations on the uniqueness of the anatomical and electrical characteristics of the PSB, PSB area pacing could be a reliable alternative for preserving interatrial synchrony with efficient anatomical access, more specific electrical and anatomy-involved capture criteria, and stable pacing lead fixation.
